# The mediating role of job satisfaction between psychological capital and work engagement among Chinese nurses during COVID-19 outbreak: A comparative study between nurse specialists and general nurses

**DOI:** 10.3389/fpsyt.2022.990216

**Published:** 2023-01-12

**Authors:** Minyi Zhang, Hongyu Chen, Ning Wang, Yao Li, Xiaofei Li, Yan Liu

**Affiliations:** ^1^Department of Neurosurgery, The First Hospital of China Medical University, Shenyang, China; ^2^Department of Transplantation/Hepatobiliary Surgery, The First Hospital of China Medical University, Shenyang, China

**Keywords:** nurses, COVID-19, psychological capital, job satisfaction, work engagement

## Abstract

**Background:**

The outbreak of COVID-19 has become a global public health emergency, causing great psychological distress to nurses. It is unknown whether the pandemic will affect the work engagement of nurses, the relationship between psychological capital, job satisfaction, and work engagement among nurses, and whether there are differences between nurse specialists and general nurses during the pandemic.

**Objectives:**

The purpose of this study was to compare psychological capital, job satisfaction, and work engagement among nurse specialists and general nurses during the pandemic, as well as to test the role of job satisfaction as a mediator in the association between psychological capital and work engagement among nurses, and to examine whether the underlying mechanism of the relationship between psychological capital and job satisfaction differs between nurse specialists and general nurses.

**Materials and methods:**

A convenience sampling was used to assess a sample of 372 nurse specialists and 318 general nurses from nine provincial general hospitals in China to participate in the online survey. Data were collected using self-report questionnaires, including the following tools: self-designed socio-demographic questionnaire, psychological capital scale, job satisfaction scale, and work engagement scale.

**Results:**

Compared with general nurses, the nurse specialists had higher psychological capital, job satisfaction, and work engagement. Job satisfaction partially mediated the positive association between psychological capital and work engagement and the indirect effect was stronger in nurse specialists in comparison to general nurses during the COVID-19 pandemic.

**Conclusion:**

The findings provide important practical implications for future intervention programs aimed at enhancing nurses’ work engagement, which may be realized through strengthening psychological capital and job satisfaction during the pandemic. Moreover, considering the cost-effectiveness of limited health care spending, nursing managers should pay more attention to the continuing professional development of young general nurses.

## Introduction

With the rapid development of health care and the aging of the population, the demand for quality care continues to increase, which brings great challenges to nursing work. However, the World Health Organization estimates that a shortfall of 5.7 million nurses has been predicted by 2030 across the world (1). In China, the number of registered nurses per 1,000 people is 2.73, which is significantly lower than in developed countries (2). The acute shortage and high turnover of nurses have become a global problem. COVID-19 has caused over 513 million confirmed cases and 6.24 million deaths worldwide, according to the World Health Organization as of May 7, 2022 (3). COVID-19 could exist for a long time, especially as the virus evolves, posing new challenges for nursing staff and potentially exacerbating the shortage of nurses in hospitals. At the same time, the pandemic had a significant negative impact on nurses’ physical and mental health, as well as their quality of life (4). Nurses who are under high pressure for a long time are prone to job burnout, which also affects the health outcomes of patients (5). Previous study showed that higher engagement results in lower intention to leave the organization and profession (6). Work engagement is a positive emotion and cognitive state related to work (7), which has been shown to help professionals cope with work-related psychological distress and contribute to their well-being and health (8), and boost job performance (9). Moreover, Bargagliotti (10) argued that in the twenty-first century, positive nurses’ work engagement is essential for nurses’ personal initiative, and for health organizations’ profitability and efficiency. Given the important role of nurses’ work engagement in nursing, investigating the level of nurses’ work engagement, which may benefit nursing managers to help nurses be more productive in responding to the ongoing pandemic.

Work engagement is a positive state of mind associated with work that is characterized by vigor, dedication, and absorption (7). Vigor refers to the willingness to invest effort in one’s work, dedication is related to participation, and absorption is related to concentration and being absorbed in one’s work (11). Work engagement keeps employees energized, passionate about solving customer problems and fully committed to their work (12). According to the Job Demands-Resources (JD-R) model of work engagement (13), work engagement is determined by two factors: job resources and personal resources. Job resources include all physical, social, psychological and organizational characteristics of a job that help people achieve goals, whereas personal resources come from individual psychological states (13). A positive psychological state manifested by an individual in the process of growth and development is known as psychological capital, which contains four dimensions of self-efficacy, optimism, hope and resilience (14). People with high self-efficacy, hope, and resilient believe that they have specific skills and resources to quickly recover from stressful situations (15–17). In addition, optimists can effectively buffer the negative effects of the pandemic (15). A previous study showed that psychological capital can maintain employee work motivation and effectively alleviate psychological stress and negative emotions, as well as job burnout (18). Employees with higher psychological capital will actively connect with other resources, and promote job satisfaction (19), and work engagement (20). However, Martin et al. (21) investigated the intention of nurses to work during the H1N1 pandemic. They found that nurses were less likely to work during pandemics if they were afraid of transmitting the infection to their family members. Therefore, psychological capital may be a useful personal resource for increasing work engagement during the pandemic. Given the positive impact of psychological capital on work, we hypothesized that psychological capital may positively affect work engagement with nurses.

Aside from psychological capital, job satisfaction has become a supportive factor in work engagement (5). Job satisfaction is usually defined as a positive and pleasant emotional reaction generated by an individual’s overall assessment (22). Previous studies have shown that job satisfaction has a positive impact on organizational commitment (23), career identity (24), job performance (19), and negative impact on turnover intention (22). At the same time, job satisfaction was a significant predictor of physical and mental health, as well as subjective well-being (25). However, the COVID-19 pandemic has increased pressure on Chinese health professionals, who have been under pressure in recent years due to the large population and increasing health awareness (26). High levels of stress and burnout are linked to lower satisfaction among nurses (27). Across health care professions and settings, job satisfaction is important because low job satisfaction of nurses has contributed to their high turnover rate and decreased quality and safety of patient care (23). The conservation of resources (COR) theory (28) states that people strive to acquire and protect resources that they find useful. Those who lack resources are not only more vulnerable to resource loss, but the initial loss also leads to future losses. Those with resources, on the other hand, are more capable of gaining, and the initial resource gain leads to additional gains. Previous researches have shown that psychological capital as an internal personal resource that helps employees respond to various work requirements with a positive psychological state, effectively prevents and improves job burnout, and finally improves their job satisfaction (19). In addition, job satisfaction as an individual’s subjective emotional state, produces a pleasant emotional response due to the realization of the individual’s work value, and the individual may be more actively engaged in work (29). That is, when individuals’ psychological and emotional needs are met and they are satisfied with their work, they may devote themselves to that work in a more active and fuller state of mind and are able to realize their work value. However, little is known about the relationship between psychological capital, job satisfaction and work engagement among nurses during the pandemic. Given the above discussion, we hypothesized that job satisfaction has a mediating effect on the relationship between psychological capital and work engagement in nurses.

In light of a potential nursing shortage, the growing burden of diseases in the aging population and scarcity of health resources sharpen the need for a sustainable nursing health human resources strategy to satisfy the rising demand for care and maximize nursing efficiency (30, 31). The development of nurse specialists has been recognized as one of the solutions to these challenges (32). A nurse specialist is described as “a nurse prepared beyond the level of a general nurse and authorized to practice as a specialist with advanced expertise in a branch of the nursing industry” by the International Council of Nurses. Clinical, educational, administration, research, and consultant roles are all part of specialized practice (33), and preventive care, chronic disease management, practice operations, nursing management, and transition care are key areas of general nurses’ practice (34). Previous researches have shown that nurse specialists’ engagement in patient care shortens hospital stays, readmissions, and emergency visits, as well as medical costs (35–37). However, it has been widely reported that nurses are facing numerous challenges during the COVID-19 pandemic, which includes an increase in workload, physical exhaustion, the need for personal protective equipment, fear of infection and infection of family members, disruption of work-life balance, and ignoring the needs of individuals and families, has put a significant deal of stress on nurses (38). It can be argued that the work engagement of nurses may be affected by occupational stress and changes in the work environment caused by the pandemic. While evaluations of nurse specialist roles have shown multiple positive outcomes in previous studies, little is known about whether nurse specialists also still have a positive impact during a pandemic.

**FIGURE 1:**
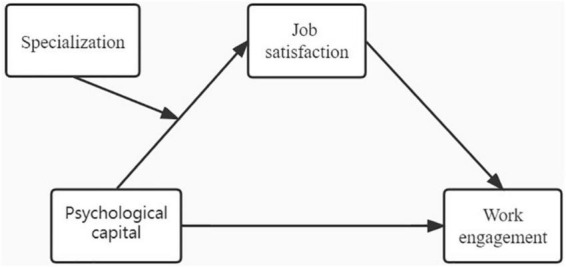
Conceptual model.

Therefore, it is necessary to focus on the impact of the pandemic on nurses’ work engagement. And because specialist nurses and general nurses have different scopes of practice, it is important to explore whether there are differences in psychological capital, job satisfaction and work engagement between them, which may benefit in promoting the continuing professional development of nursing staff. In addition, little is known about the relationship between psychological capital, job satisfaction and work engagement among nurses during the pandemic. As a result, the purpose of our study is to compare psychological capital, job satisfaction, and work engagement among nurse specialists and general nurses during the pandemic, as well as propose the hypotheses that the relationship of psychological capital on work engagement is mediated by job satisfaction, and to test whether the underlying mechanism of the relationship between psychological capital and job satisfaction differs between nurse specialists and general nurses during the COVID-19 pandemic. A moderated mediation model (Figure 1) was constructed to address the hypotheses that the effect of psychological capital on work engagement was mediated by job satisfaction and moderated by specialization. Considering the positive assessment of the nurse specialist roles in previous studies (35–37), we hypothesized that the association between psychological capital and job satisfaction would be strengthened for nurse specialists during COVID-19 pandemic. Specifically, the relationship between psychological capital and job satisfaction might be more powerful in nurse specialists than general nurses.

## Materials and methods

### Design, setting, and participants

A multi-center cross-sectional survey was carried out from May to October of 2021 from 9 provinces in China. The researchers created an electronic questionnaire on the questionnaire star platform and sent it to nursing administrators in nine provincial general hospitals of China through WeChat, mainly including Liaoning, Jilin, Heilongjiang, Sichuan, Guangdong, Anhui, Henan, Anhui, Guangxi Province. They were asked to deliver the survey to the nurse through We Chat. Participants could scan the QR to read and submit the informed consent agreement and questionnaires. Each participant was only allowed to submit once in order to avoid double submission.

The following were the eligibility criteria for nurse specialist enrollment: (a) hold a Chinese nursing specialist certificate after completing at least 6 months of training; (b) hold a Chinese registered nurse license; (c) work full-time; (d) have submitted a declaration of consent.

The following eligibility criteria for general nurse were: (a) hold a Chinese registered nurse license; (b) work full-time; (c) have submitted a declaration of consent.

The exclusion criteria for two group nurses were nursing staff who were not directly involved in patient care, such as absent due to sick leave, personal leave, study, or further training.

The sample size calculator G*Power (3.0.10) was used (39). To identify a mean difference (two-tail) with a 50% effect size, 5% estimated error and 95% power (1–β), 210 participants were required. To account for 20% attrition, a total of 252 participants were required (126 in each group). The study recruited 690 nurses from nine provincial general hospitals in China, which included 372 nurse specialists and 318 general nurses.

### Variables and measurements

#### Social-demographic questionnaire

According to the purpose of our study, we designed the general socio-demographic data, which mainly includes gender, age, health status, physical condition, marital status, family status, years of working, education, job title, department position, total night shifts per month and monthly income.

#### Psychological capital scale

The Chinese version of the Psychological Capital Scale was used to assess the psychological capital of nurses. Luthans et al. (40) developed the scale and the Chinese scholar Luo (41) revised it based on the characteristics of nursing work. It contains 20 items and four dimensions, which include self-efficacy (6 items), hope (6 items), resilience (5 items), and optimism (3 items). The scale used the six-point Likert, with a total score of 20 to 120 points, ranging from “strongly disagree” (1 point) to “strongly agree” (6 points). The greater the psychological capital, the higher the score. The total Cronbach’s alpha was 0.92 and each subscale of Cronbach’s alpha ranged from 0.88 to 0.95 (41). In our study, the scale’s Cronbach’s alpha was 0.91, and the Cronbach’s alpha of each subscale was 0.89, 0.93, 0.92, and 0.88.

#### Job satisfaction scale

The job satisfaction scale of medical staff developed by Chinese scholar Wang et al. (42) was used to assess job satisfaction among nurses in our study. It primarily consists of 20 entries and six dimensions: work itself (2 items), work pressure (2 items), interpersonal relationship (4 items), working condition (4 items), work return (4 items), and organizational management (4 items). All items were rated on a five-point Likert scale ranging from 1 (Strongly disagree) to 5 (Strongly agree). This scale has revealed good reliability and validity. The total score on the scale ranges from 20 to 100, with a higher total score indicating a higher level of job satisfaction. The total Cronbach’s alpha was 0.91 and each subscale of Cronbach’s alpha ranged from 0.74 to 0.89 (42). In this study, the total Cronbach’s alpha was 0.90, and the Cronbach’s alpha of each domain was 0.74, 0.80, 0.85, 0.88, 0.89, and 0.83.

#### Utrecht work engagement scale (UWES-9)

The Chinese version of the Utrecht Work Engagement Scale was used to assess work engagement of nurses. It was developed by Schaufeli et al. (7) with nine items. It consists of three subscales: vigor (3 items), dedication (3 items), and absorption (3 items). Each item is given a seven-point Likert scale from 0 (never) to 6 (always), with a total score of 0 to 54. A higher score indicates a higher level of work engagement. The total Cronbach’s alpha was 0.93 and each subscale of Cronbach’s alpha ranged from 0.81 to 0.90 (7). The scale has good reliability and validity. In this study, the total Cronbach’s alpha was 0.94, and the Cronbach’s alpha of each domain was 0.82, 0.93, and 0.83.

### Data collection

To ensure accuracy, two researchers who did not know the study design entered the data after all surveys were completed. They received training on how to check, input and code the data into IBM SPSS v23.0 before analyzing the data. Only if they pass the training exam can they participate in data entry.

### Data analysis

The IBM SPSS v23.0 software was used to analyze the data. First, continuous data were represented by mean and standard deviation, and categorical data or rank data were represented by frequency or percentage. The Chi-square test was used to compare differences in general demographic characteristics between groups. Confirmatory factor analysis was used to check for common method variance (43). Second, the *t*-test was used to analyze and compare the psychological capital, job satisfaction, and work engagement of the nurse specialist group and the non-nurse specialist group. Third, Pearson’s or Spearman’s correlation analysis was used to analyze the correlation between social-demographic variables, psychological capital, job satisfaction and work engagement among nurses. To check for collinearity among the independent variables, both bivariate Pearson’s correlations and variance inflation factors were performed, with significant correlations less than 0.8 among these variables and variance inflation factors (VIFs) were < 10, indicating that multicollinearity was not a problem. Then, a simple mediation analysis of job satisfaction mediating the relationship between psychological capital and work engagement was tested using Hayes’s PROCESS macro for SPSS (Model 4) (44). The indirect effect of mediation was tested using a bootstrapping method with 5,000 samples as recommended, with a significant effect indicated by a 95% confidence interval not including zero. Finally, PROCESS macro (Model 7) (44) was utilized to examine the moderated mediation model, followed by a simple slope test (45).

According to the previous studies (46), age, sex, years of nursing experience, night shift, professional practice environment and professional status were found to predict work engagement of registered nurse. This mediation analysis was controlled for relevant social-demographics of nurses. In the current study, *P*-value was two-tailed and we inferred statistical significance if α was < 0.05.

## Results

### Common method variance (CMV) test

The data for this study were collected through self-report, which may have been influenced by common method bias, reducing the validity of the results. In this study, we followed strict confidentiality and voluntarism principles and asked participants to be honest in their answers to each question. Data collectors are used to collect and enter data. These methods effectively control the bias of the common method. In addition, following the method suggested by Podsakoff et al. (43), common method variance was tested by controlling for the effects of an unmeasured latent factor. Confirmatory factor analysis was used to test the common method bias of all self-assessment items. The results showed that the model fit was poor, χ^2^ = 2686.43, degrees of freedom (df) = 170, χ^2^/*DF* = 15.803, comparative fit index (CFI) = 0.849, goodness of fit (GFI) = 0.676, normed fit index (NFI) = 0.841, root mean square error of approximation (RMSEA) = 0.147, which indicating that there is no significant common method variance problem in this study.

### Demographic characteristics of the participants

A total of 700 nurses took part in the study. After excluding 10 invalid questionnaires, a total of 690 valid questionnaires remained for an completion rate of 98.6%, which included 372 nurse specialists and 318 general nurses. Females predominated in both groups. In terms of age, 206 (55.4%) of nurse specialists were over 35 years old, while only 59 (18.56%) of general nurses were, indicating that most numbers of general nurses were younger than nurse specialists. The age difference between the two groups was significant (*p* < 0.001). In nurse specialists, 80.11% had children compared to 49.37% in general nurses, respectively (Table 1, *p* < 0.001). 138 (37.1%) of nurse specialists had worked between 11 and 15 years, 104 (27.96%) had worked more than 21 years, and 86.48% of general nurses had worked fewer than 15 years. In terms of working years, there was a considerable difference between the two groups (*p* < 0.001). Among nurse specialists, 2.69% were junior nurses, 26.61% were senior nurses, 52.15% were nurses-in-charge and 18.55% were vice-director nurses and above, however, 18.90% were junior nurses, 51.90% were senior nurses, 23.90% were nurses-in-charge and 5.30% were Vice-director nurse and above in general nurses. There was a significant difference in the job title between the two groups (*p* < 0.001). Among the department position of nurse specialists and general nurses, there were 155 (41.67%) and 246 (77.36%) of general nurses, 55 (14.78%) and 27 (8.49%) of team teachers, 49 (13.17%) and 17 (5.35%) of nursing team leaders, 99 (26.61%) and 25 (7.86%) of head nurses, and 14 (3.76%) and 3 (0.94%) head nurses and above. There was a significant difference in department position, marital status and the number of night shifts per month between the two groups (*p* < 0.001), but no difference in educational level, health status and monthly income (*p* = 0.227).

**TABLE 1:** Differences in general characteristics between nurse specialists and general nurses.

Demographic characteristics	Nurse specialist (*n* = 372)	General nurses (*n* = 318)	Total (*n* = 690)	χ^2^	*P*
Gender				7.432	0.006**
Male	15 (4.03%)	29 (9.12%)	44 (6.38%)		
Female	357 (95.97)	289 (90.88%)	646 (93.62%)		
Age				187.325	<0.001***
≤25	3 (0.80%)	42 (13.20%)	45 (6.52%)		
26˜30	32 (8.60%)	134 (42.14%)	166 (24.06%)		
31˜35	131 (35.22%)	83 (26.10%)	214 (31.01%)		
36˜40	92 (24.73%)	33 (10.38%)	125 (18.12%)		
≥41	114 (30.65%)	26 (8.18%)	140 (20.29%)		
Physical condition				2.354	0.502
Poor	6 (1.60%)	5 (1.57%)	11 (1.59%)		
General	120 (32.30%)	115 (36.16%)	235 (34.06%)		
Better	175 (47.00%)	150 (47.17%)	325 (47.10%)		
Very good	71 (19.10%)	48 (15.09%)	119 (17.25%)		
Marital status				47.735	<0.001***
Single	50 (13.44%)	114 (35.85%)	164 (23.77%)		
Married	310 (83.33%)	198 (62.26%)	508 (73.62%)		
Divorced	12 (3.23%)	6 (1.89%)	18 (2.61%)		
Family status				78.701	<0.001***
Do not have children	74 (19.89%)	161 (50.63%)	235 (34.06%)		
Has children	298 (80.11%)	157 (49.37%)	455 (65.94%)		
Yeas of working				197.035	<0.001***
≤1 year	0 (0%)	16 (5.03%)	16 (2.32%)		
2˜5 years	13 (3.49%)	111 (34.91%)	124 (17.97%)		
6˜10 years	52 (13.98%)	82 (25.79%)	134 (19.42%)		
11˜15 years	138 (37.1%)	66 (20.75%)	204 (29.57%)		
16˜20 years	65 (17.47%)	15 (4.72%)	80 (11.59%)		
≥21 years	104 (27.96%)	28 (8.81%)	132 (19.13%)		
Education				6.835	0.077
Below university	6 (1.61%)	3 (0.94%)	9 (1.30%)		
College	28 (7.53%)	41 (12.89%)	69 (10.00%)		
Undergraduate	332 (89.25%)	266 (83.65%)	598 (86.67%)		
Master’s degree and above	6 (1.61%)	8 (2.52%)	14 (2.03%)		
Job title				131.808	<0.001***
Junior nurse	10 (2.69%)	60 (18.90%)	70 (10.15%)		
Senior nurse	99 (26.61%)	165 (51.90%)	264 (38.26%)		
Nurse-in-charge	194 (52.15%)	76 (23.90%)	270 (39.13%)		
Vice-director nurse and above	69 (18.55%)	17 (5.30%)	86 (12.46%)		
Department position				93.352	<0.001***
Ordinary nurse	155 (41.67%)	246 (77.36%)	401 (58.12%)		
Team teacher	55 (14.78%)	27 (8.49%)	82 (11.88%)		
Team leader	49 (13.17%)	17 (5.35%)	66 (9.57%)		
Head nurse	99 (26.61%)	25 (7.86%)	124 (17.97%)		
Head nurse and above	14 (3.76%)	3 (0.94%)	17 (2.46%)		
Total night shifts per month				69.983	<0.001***
0	166 (44.62%)	76 (23.90%)	242 (35.07%)		
1˜2	73 (19.62%)	28 (8.81%)	101 (14.64%)		
3˜4	16 (4.30%)	34 (10.69%)	50 (7.25%)		
5˜6	50 (13.44%)	70 (22.01%)	120 (17.39%)		
>6	67 (18.01%)	110 (34.59%)	177 (25.65%)		
Monthly income (RMB)				6.220	0.101
<2,000	1 (0.27%)	4 (1.26%)	5 (0.73%)		
2,000˜5,000	109 (29.30%)	86 (27.04%)	195 (28.26%)		
5,000˜10,000	195 (52.42%)	152 (47.80%)	347 (50.29%)		
>10,000	67 (18.01%)	76 (23.90%)	143 (20.73%)		

*p < 0.05, **p < 0.01, ***p < 0.001.

As shown in Table 2, the nurse specialists had a higher psychological capital score (95.52 ± 15.83) than general nurses (92.12 ± 17.70). The nurse specialists scored higher than general nurses on the four dimensions of self-efficacy, hope, resilience and optimism (*p* < 0.05). The nurse specialists’ job satisfaction score (76.59 ± 12.20) was significantly higher than general nurses (74.44 ± 12.57). In terms of work itself, interpersonal relationship, and work return, the nurse specialists scored significantly higher than general nurses (*p* < 0.05). However, there was no significant difference in work pressure, working condition, and organizational management between the two groups (*p* > 0.05). The nurse specialists’ work engagement scores (39.22 ± 12.35) and vigor scores (13.20 ± 4.15) were significantly higher than general nurses (37.16 ± 12.65) and (12.21 ± 4.44). However, we found no statistically significant differences in dedication and absorption scores between the two groups (*p* > 0.05).

**TABLE 2:** Comparison of psychological capital, job satisfaction, and work engagement scores between nurse specialists and general nurses.

Variables	Nurse specialists (*n* = 372) M ± SD	General nurses (*n* = 318) M ± SD	*t*	*P*
Psychological capital	95.52 ± 15.83	92.12 ± 17.70	−2.974	0.003**
Self-efficacy	28.99 ± 4.93	27.65 ± 5.69	−3.325	0.001**
Hope	28.23 ± 5.00	27.19 ± 5.71	−2.572	0.010*
Resilience	24.06 ± 4.22	23.23 ± 4.48	−2.484	0.013*
Optimism	14.63 ± 2.70	14.05 ± 2.91	−2.719	0.007**
Job satisfaction	76.59 ± 12.20	74.44 ± 12.57	−2.276	0.023*
Work itself	7.65 ± 1.68	7.30 ± 1.76	−2.649	0.008**
Work pressure	7.12 ± 1.61	7.09 ± 1.73	−0.284	0.777
Interpersonal relationship	16.49 ± 2.52	15.79 ± 2.72	−3.495	0.001**
Working condition	15.15 ± 3.22	14.93 ± 3.20	−0.895	0.371
Work return	14.72 ± 3.18	14.23 ± 3.11	−2.035	0.042*
Organizational management	15.47 ± 3.25	15.11 ± 3.25	−1.464	0.144
Work engagement	39.22 ± 12.35	37.16 ± 12.65	−2.154	0.032*
Vigor	13.20 ± 4.15	12.21 ± 4.44	−3.001	0.003**
Dedication	12.86 ± 4.53	12.20 ± 4.73	−1.886	0.063
Absorption	13.16 ± 4.37	12.75 ± 4.30	−1.248	0.213

*p < 0.05, **p < 0.01, ***p < 0.001.

Table 3 shows the correlations, mean and standard deviations of the three variables in the study (*n* = 690). The score for work engagement was 38.27 (SD = 12.52). The scores for psychological capital and job satisfaction were 94.17 (SD = 16.82) and 75.60 (SD = 12.41), respectively. As shown in Table 3, the result of Pearson’s correlation analysis revealed that psychological capital and job satisfaction were positively associated with work engagement (*r* = 0.776, *p* < 0.001; *r* = 0.629, *p* < 0.001). Moreover, psychological capital was found to be positively related to job satisfaction (*r* = 0.704, *p* < 0.001). The absolute values of the correlation coefficients between the three variables were less than 0.8, indicating a weak to moderate correlation between the variables and no multicollinearity problem (47).

**TABLE 3:** Correlations, means, standard deviations of variables among nurses (*n* = 690).

Variables	M ± SD	1	2	3
1. Psychological capital	94.17 ± 16.82	1		
2. Job satisfaction	75.60 ± 12.41	0.704***	1	
3. Work engagement	38.27 ± 12.52	0.776***	0.629***	1

***p < 0.001.

Table 4 shows the correlation analysis of the variables between nurse specialists and general nurses. The results revealed that age, physical condition, years of working, job title, and department position were all positively associated with psychological capital, job satisfaction and work engagement, while total night shifts per month was negatively associated with all three variables among nurse specialists (*p* < 0.05). Moreover, gender, years of working were all positively related to psychological capital, job satisfaction and work engagement, while total night shifts per month were negatively related to all three variables among general nurses (*p* < 0.05). In addition, the findings also showed positive correlations between psychological capital, job satisfaction, and work engagement in both groups (*p* < 0.001).

**TABLE 4:** Correlation analysis of variables between nurse specialists and general nurses.

Variables	Nurse specialists (*n* = 372)			General nurses (*n* = 318)		
	**Psychologic-al capital**	**Job satisfaction**	**Work engagement**	**Psychologi-cal capital**	**Job satisfaction**	**Work engagement**
1. Gender	0.049	0.079	0.079	0.135**	0.121**	0.114**
2. Age	0.170***	0.140**	0.110**	0.095	0.277***	0.088
3. Physical condition	0.175***	0.128**	0.255***	0.217***	0.05	0.183***
4. Marital status	0.064	0.033	0.102**	0.102	0.048	0.112**
5. Family status	0.006	-0.009	0.051	0.098	-0.019	0.113**
6. Yeas of working	0.120**	0.188***	0.209***	0.121**	0.125**	0.121**
7. Education	0.007	0.023	0.034	0.082	0.041	0.095
8. Job title	0.110**	0.160***	0.215***	0.123**	0.065	0.169***
9. Department position	0.038	0.175***	0.196***	0.129**	0.003	0.134**
10. Total night shifts per month	-0.112**	-0.331***	-0.136***	-0.212***	-0.146***	-0.25**
11. Monthly income	0.079	0.169***	0.048	0.037	0.005	0.119**
12. Psychological capital	1	-	-	1	-	-
13. Job satisfaction	0.711***	1	-	0.683***	1	-
14. Work engagement	0.751***	0.644***	1	0.780***	0.648***	1

***p < 0.001, **p < 0.05.

### Testing for mediating effects among nurses (*n* = 690)

According to the Hypothesis, we investigated if the relationship between psychological capital and work engagement would be mediated by job satisfaction (Table 5). Before considering the mediating role of job satisfaction, we initially investigated the main effect of psychological capital on work engagement and discovered that psychological capital was positively associated with work engagement (β = 0.771, *p* < 0.001). The mediating effect was then investigated using model 4 in the PROCESS macro. After accounting for nurses’ socio-demographic, psychological capital was found to be positively associated with job satisfaction (β = 0.715, *p* < 0.001), which in turn predicted work engagement (β = 0.177, *p* < 0.001). Job satisfaction also had a significant indirect effect [indirect effect = 0.126, SE = 0.025, 95% CI = (0.080, 0.176)], according to bootstrapping analyses. Furthermore, there was a significant direct relationship between psychological capital and work engagement (β = 0.645, *p* < 0.001). Therefore, job satisfaction partially mediated the relationship between psychological capital and work engagement. The mediating effect accounted for 16.34% of the total effect.

**TABLE 5:** Testing the mediation effect of psychological capital on work engagement *via* job satisfaction (*N* = 690).

Predictors	Model 1 (work engagement)		Model 2 (job satisfaction)		Model 3 (work engagement)		Indirect effect of job satisfaction			
	**β**	**t**	**β**	**t**	**β**	**t**				
							**Indirect effect**	**SE**	**LLCI**	**ULCI**
Psychological capital	0.771***	31.118	0.715***	25.729	0.645***	18.883	0.176	0.025	−0.080	0.176
Job satisfaction					0.177***	5.267				
*R* ^2^_*adj*_	0.607		0.507		0.623					
*F*	116.800***		77.574***		112.027***					

Each column represents a regression on the model, which predicts the criterion at the top of the column. All models are adjusted for gender, age, marital status, family status, yeas of working, job title, department position, and total night shifts per month.

***p < 0.001.

### Testing for moderated mediation

The study hypothesized that specialization would moderate the effect of psychological capital on job satisfaction. As shown in Table 6, the interaction between psychological capital and job satisfaction had a significant effect on work engagement (β = 0.301, *p* < 0.05), indicating that the effect of psychological capital on job satisfaction was moderated by specialization. Thus, the moderated mediation model was established as the first stage of the mediation effect was moderated by specialization.

**TABLE 6:** Testing for moderated mediation effect (*N* = 690).

	β	SE	LLCI	ULCI
**Mediator variable model (Outcome: Job satisfaction)**				
Psychological capital	0.716***	0.028	0.663	0.772
Specialization	0.268	0.031	0.000	0.123
Psychological capital × Specialization	0.301**	0.027	0.002	0.108
**Dependent variable model (Outcome: Work engagement)**				
Psychological capital	0.645***	0.034	0.578	0.712
Job satisfaction	0.177***	0.034	0.111	0.243
**Conditional indirect effect analysis**				
Nurse specialists	0.202	0.027	0.086	0.192
General nurses	0.173	0.024	0.072	0.167
Index of moderated mediation	0.029	0.011	0.020	0.045

Each column represents a regression on the model, which predicts the criterion at the top of the column. All models are adjusted for gender, age, marital status, family status, yeas of working, job title, department position, and total night shifts per month.

***p < 0.001, **p < 0.05.

**FIGURE 2:**
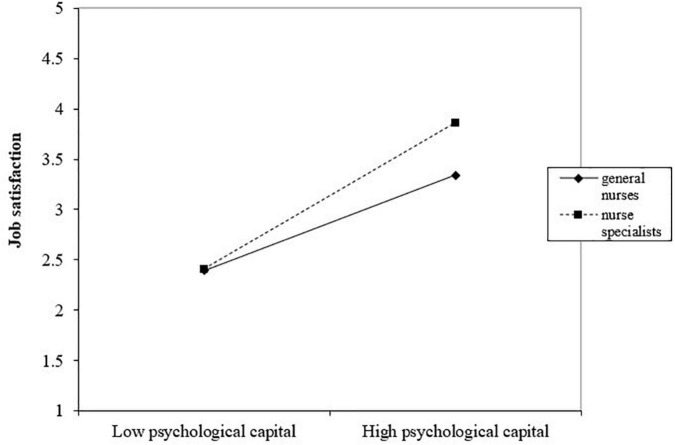
Specialization as a moderator of the association between psychological capital and job satisfaction.

The results of the simple slope analysis revealed that psychological capital was significantly positively associated with job satisfaction for nurse specialists (β_*simple*_ = 0.866, *p* < 0.001), whereas for general nurses, the association between psychological capital and job satisfaction was still significant, but much smaller (β_*simple*_ = 0.566, *p* < 0.001). The study plotted the association of psychological capital with job satisfaction separately for nurse specialists and general nurses to interpret interactions with moderating variables (Figure 2). As shown in Figure 2, psychological capital had less impact on job satisfaction when specialization was low, and the relationship was strengthened when specialization was high. Thus, our hypothesis was supported.

Table 6 also showed the conditional indirect effect of psychological capital on work engagement. The result indicated that the index of moderated mediation is significant [β = 0.029, 95% CI = (0.020, 0.045)]. Specifically, the indirect effect of psychological capital on work engagement *via* job satisfaction was stronger for nurse specialists [β = 0.202, 95% CI = (0.086, 0.192)] than for general nurses [β = 0.173, 95% CI = (0.072, 0.167)].

## Discussion

This study compared the work engagement, psychological capital, and job satisfaction of nurse specialists and general nurses and to investigate the relationship between psychological capital and work engagement of nurses, as well as the role of job satisfaction in mediating the relationship between psychological capital and work engagement in China during the pandemic. We found that compared with general nurses, nurse specialists had higher work engagement, psychological capital and job satisfaction, psychological capital was positively correlated with work engagement. Job satisfaction partially mediated the positive association between psychological capital and work engagement. Specifically, the association between psychological capital and job satisfaction was stronger for nurse specialists compared to general nurses during the COVID-19 pandemic. Overall, the results explain the hypotheses proposed in this study.

The study found that nurses showed a higher level of work engagement during the COVID-19 pandemic than Wan et al. (48) were before the pandemic. This may be due to the fact that the Chinese government established very specific national rules and regulations to prevent the spread of the virus and enforced them from the central to local government strictly and consistently (49), with proactive strategies like enhancing the public health system, securing the supply of pandemic prevention and control equipment, and conducting pandemic prevention training (50). In addition, the Chinese government in a collective way has encouraged citizens to form a shared sense of responsibility, emphasized group interests, and advocated actions for the common good (51). As a result, nurses are more confident in dealing with pandemic, addressing their fears and demonstrating a strong sense of moral obligation, vocation and values associated with the professions (52). Individuals with a strong vocation are more likely to commit to their profession because they perceive their personal mission more clearly, are more focused on their goals, and have a clear sense of meaning and identity, which may promote the development of work engagement (53).

The study found that nurse specialists had a higher level of work engagement than general nurses. This could be because the two groups of nurses differed in age, years of working, night shift, professional practice environment, and professional status (such as job title or department position), which was consistent with the previous study (46). Vigor received the highest score among nurse specialists. It could be explained the fact that most nurse specialists have senior professional titles, extensive clinical and management experience, and a stronger feeling of responsibility and mission in the field of nursing work, which leads to higher work engagement. People with a sense of mission are willing to face challenges and difficulties, and even make sacrifices for them, even in a stressful work environment (54). Moreover, when employees believe that their work can create value and make sense to themselves, they have a higher sense of career identity, more engagement and satisfaction with their work (24). However, the highest score in general nurses was absorption, possibly due to most nurses with a low job title and young, and a lack of relevant clinical experience to combat the pandemic, which were more likely to experience more fatigue (55), so that they need to spend more time and energy dealing with work. The previous research on avian influenza A/H7N9 has shown that medical personnel with less than 5 years of experience or without relevant training and experience are more likely to experience psychological problems (56). Therefore, nursing managers must provide more protective psychological intervention to recover their mental health and help them improve professional knowledge and skills, strengthen their career identity and sense of responsibility, and thus improve their work engagement.

We found that the psychological capital of nurse specialists was significantly higher than that of general nurses. On the four dimensions of self-efficacy, hope, resilience and optimism, the nurse specialists outperformed the general nurses. It could be due to the fact that self-efficacy is one’s belief in their ability to achieve their goals (14), which can control personal emotions and their management, and improve individuals’ psychological well-being and mental health (16). In our study, higher level of self-efficacy among nurse specialists suggest that they may have adequate abilities to cope with the negative impact of the pandemic, maintaining relatively stable emotions even under pressure, confirming the important role of self-efficacy in adaptive confrontation styles during the pandemic. This result is consistent with previous studies that self-efficacious individuals have positive attitudes toward life and are psycho-emotionally able to cope with difficult situations, changes, and stress (16). Second, hope is a positive motivation state based on the inner sense of success, that is, the way and plan to achieve the goal (57). According to prior research (24), a high level of hope may enable nurses to effectively deal with psychological distress and cope with difficulties at work more positively, and drive them to pursue professional development. Our findings support previous research that hope play an important role in the face of the uncertainty that characterizes the current work and career environment during the pandemic (58). Third, optimism was highest among the two groups, which shows that nurses are coping positively with the negative effects of the pandemic. This result is consistent with previous studies that optimism seems to moderate the effect of stressful life events on depression and anxiety symptoms (59). Furthermore, resilience is defined as an individual’s ability to successfully cope with adversity, resist illness, and adapt to new situations in order to maintain psychological health (60). Highly resilient healthcare workers may have adequate coping resources and positive emotions, can effectively address COVID-19-related stress and withstand the pandemic-related psychological burden, thereby reducing the negative outcomes related to the job (61, 62). These findings are consistent with those of previous studies (15), confirming that nurse specialists high in psychological capital may be more likely to overcome adversity and deal effectively with potential stressors during the pandemic. Therefore, nursing managers should pay attention to the psychological capital of general nurses. This is cost-effective, since training nurses is costly and resources can be directed from being wasted efforts to productive activities (63). Nurse managers can provide effective interventions by holding mindful activities that stimulate positive emotions (64), deliver psychological counseling lectures (65), and conduct resilience training (17) to reduce nurses’ psychological pressures and negative emotions during the pandemic.

The study found that the job satisfaction of nurse specialists was higher than general nurses. This could be due to the fact that general nurses are mostly young nurses who had to shoulder the responsibility for coping with the pandemic in addition to their regular nursing duties. A previous study reported that of the 28,600 nurses recruited from across China to provide care to patients with COVID-19 in Hubei, 40% were younger than 30 years, which indicated young health-care providers were the backbone of the COVID-19 crisis in China (66). Therefore, their work is likely to be more intensive and stressful, which may lead to lower job satisfaction. Previous studies have shown that heavy workloads have a negative impact on job satisfaction (22). Moreover, most of them are new nurses who lack a definite career path, frequently work night shifts, and have lower stress resistance and coping strategies, all of which may reduce job satisfaction. On the contrary, as working years and experience accumulate, nurse specialists gradually gain decision-making power in their work, and it is easier to gain a sense of accomplishment and career identity (67). Scanlan et al. (68) demonstrated that career identity facilitates employees to effectively obtain the required work resources, assists to create a comfortable working atmosphere, so as to enhance employees’ job satisfaction and reduce employees’ turnover intention. Therefore, nursing managers can improve the job satisfaction of general nurses by providing skills training, enhancing career identity and nursing values to guide their career path, and rationalizing shift work during the pandemic.

A previous study noted that experienced nurses were appreciated by students and young nurses for the knowledge and skills that helped ease their transition to the workplace, allowing them to better realize their roles within the healthcare team and their respective scopes of practice (69). However, one of the most frequently identified challenges in the nurse specialist profession is a lack of recognition by position and title (70). One way to recognize the importance of these nurse specialists is to actively engage them in the professional development of students and young nurses. This will be of great benefit to health care organizations by ensuring sufficient training and mentorship for novice nurses while helping to establish a cohesive health care team during the pandemic. In addition, nursing managers can take advantage of clinical ladder plans to provide a training framework for nurses to promote their continued professional development (71).

One of the key findings of the study was that psychological capital was positively associated with work engagement, which could be explained by the JD-R model theory (13) stated that work engagement would persist if an individual had enough job and personal resources to complete the demanding tasks. This finding was consistent with those of previous studies (18, 20). Therefore, nurses with higher psychological resource can effectively deal with stressful events, thereby promote their work engagement during the pandemic. Moreover, job satisfaction partially mediated the positive association between psychological capital and work engagement during the pandemic. The mediating effect accounted for 16.34% of the total effect (Figure 1). This result suggests that the underlying mechanism between psychological capital and work engagement can be explained by job satisfaction, which supports the COR theory’s (28) predictions that persons with more psychological capital also have more positive psychological resources, which helps them stay motivated and prevent burnout at work, and these positive psychological traits promote their job satisfaction and may stimulate them to strive and focus on their work. The results of this study underscore the importance of job satisfaction in increasing nurses’ work engagement during ongoing pandemics. A previous studies reported (72), in a large UK study conducted in April 2020, 60% of nurses reported being professionally dissatisfied and demoralized. Therefore, in the context of the COVID-19 pandemic, nursing managers should focus on cultivating nurses’ positive psychological capital (73), leading nurses to actively respond to work pressure and enhancing nurses’ work engagement to work by creating a positive and supportive working environment. In addition, the results of the moderated mediation model also revealed that the specialization moderated the indirect impact of psychological capital on work engagement through job satisfaction among nurses during the pandemic. Specifically, the association between psychological capital and job satisfaction was stronger for nurse specialists in comparison to general nurses during the COVID-19 pandemic. This could be explained by the fact that the COVID-19 crisis has challenged existing roles and shifted organizational priorities and staff responsibilities (74). Most nurse specialists with high psychological capital and years of experience played an important role in the planning, training, and evaluation of crisis preparation, which adapted to the needs of the organization and expanded their responsibilities to provide crisis leadership that instilled calmness, confidence, trust, and resiliency in the staff during the COVID-19 pandemic (74), making it easier to gain a sense of career identity and improve job satisfaction (67, 68),which may boost their work engagement. Therefore, more attention should be paid to the continuing professional development of young general nurses to equip them with the necessary professional knowledge and ability (75). It could also develop relevant reward systems and provide promotion opportunities for outstanding performance during the pandemic (51), promote a sense of safety, and support a shared learning to meet nurses’ needs for self-realization, improve their job satisfaction, thereby improving their work engagement.

### Limitations and recommendations for future studies

Although the present study compared the work engagement between two groups of nurses and furthers our understanding of the mediating mechanism underlying the association between psychological capital and work engagement among Chinese nurses, several limitations need to be acknowledged. First, because this is a cross-sectional study, the causality of the associations between psychological capital, job satisfaction and work engagement cannot be inferred. However, we believe that our findings still provide useful and important information on the mediation model between psychological capital and work engagement. Future longitudinal study designs are needed to more robustly validate the causal relationship in this model. Second, the study data were derived from self-reports, which could lead to potential subjective and recall bias that could affect the accuracy of the assessment. Moreover, social expectations bias among participants should be considered. In this study conducted in the same hospitals setting, nurses may likely report similar work engagement results, as they felt it might make others feel that they had high levels of work engagement, particularly during the pandemic. To reduce this bias, researchers reassured all participants that there were no right or wrong answers and were informed to answer all questions based on their first instinct to minimize the impact of elucidating socially desirable responses. In addition, all participants were recruited from nine provinces in China. Therefore, the results may not apply to all nurses. Long-term studies that require random sampling in other organizational and cultural contexts should be considered to improve the generalizability of the results in the future.

As evidence has shown that mental health support is urgently needed to help nurses be more productive all over the world in combating the COVID-19 pandemic to relieve their psychological distress (76). Our studies suggest that increasing psychological capital and job satisfaction among nurses may be an effective and useful way to improve their work engagement under the COVID-19 pandemic.

## Conclusion

Our results suggest that compared with general nurses, nurse specialists had higher work engagement, psychological capital, and job satisfaction. More importantly, this study is primarily based on the JD-R model and considers the COR theory to explore that job satisfaction partially mediated the positive association between psychological capital and work engagement, and the indirect effect was stronger in nurse specialists in comparison to general nurses during the COVID-19 pandemic. Our study provides nursing managers with suggestions for team building and management that have theoretical and practical significance. Consider the cost-effectiveness of limited healthcare expenditures during the pandemic, nursing managers should pay more attention to the continuing professional development of young general nurses, ensuring sufficient training and mentorship for them in combating the pandemic by experienced nurse specialists, which helps to create a cohesive health care team during the pandemic. Second, our finding can help nursing managers correctly understand the mechanism of the relationship among psychological capital, job satisfaction and work engagement and adopt effective intervention strategies to promote nurses’ work engagement.

## Data availability statement

The original contributions presented in this study are included in the article/supplementary material, further inquiries can be directed to the corresponding author.

## Ethics statement

The studies involving human participants were reviewed and approved by the First Affiliated Hospital of China Medical University (approval number: [2020]194). The patients/participants provided their written informed consent to participate in this study.

## Author contributions

MZ and YLiu conceptualized and designed the project. MZ, HC, NW, and YLi acquired and managed the data and performed statistical and data analysis. MZ and HC drafted the manuscript. YLiu and XL revised the manuscript. All authors contributed to the article and approved the submitted version.
